# Risk factors associated with disease severity and length of hospital stay in COVID-19 patients

**DOI:** 10.1016/j.jinf.2020.04.008

**Published:** 2020-07

**Authors:** Xiaofan Liu, Hong Zhou, Yilu Zhou, Xiaojun Wu, Yang Zhao, Yang Lu, Weijun Tan, Mingli Yuan, Xuhong Ding, Jinjing Zou, Ruiyun Li, Hailing Liu, Rob M. Ewing, Yi Hu, Hanxiang Nie, Yihua Wang

**Affiliations:** a Department of Pulmonary and Critical Care Medicine, The Central Hospital of Wuhan, Tongji Medical College, Huazhong University of Science and Technology, Wuhan, Hubei, China; b Biological Sciences, Faculty of Environmental and Life Sciences, University of Southampton, Southampton SO17 1BJ, UK; c Institute for Life Sciences, University of Southampton, Southampton SO17 1BJ, UK; d Department of Respiratory & Critical Medicine, Renmin Hospital of Wuhan University, Wuhan 430060, Hubei, China; e NIHR Southampton Biomedical Research Centre, University Hospital Southampton, Southampton SO16 6YD, UK

*Dear Editor,*

We read with interest the article in this journal which revealed the critical role of timely supply of medical resources for COVID-19 patients.^1^ The pandemic of COVID-19 has placed an enormous burden on health authorities across the world. The virus, severe acute respiratory syndrome coronavirus 2 (SARS-CoV-2; previously known as 2019-nCoV), causes acute respiratory disease with common signs of infection being respiratory symptoms, fever, cough and breathing difficulties. In more severe cases, infection causes pneumonia, lung failure, septic shock, organ failure and risk of death. The WHO reports that 80% of those infected will develop mild symptoms, 14% severe symptoms and 6% will become critically ill. Given the wide clinical spectrum of COVID-19, a key challenge faced by frontline clinical staff is prioritisation of stretched resources. Thus, there is a critical need for robust risk assessment for clinical management.

To address this, we identified consecutive patients with moderate or severe COVID-19 discharged from the general wards of Renmin Hospital of Wuhan University between 5 February 2020 to 14 March 2020 (Ethics approval No: WDRY2020-K124). All patients had been diagnosed with COVID-19 according to WHO interim guidance and had radiologic evidence of pneumonia or infiltrates on chest CT scan according. The criteria for patient discharge was the absence of fever for at least 3 days, substantial improvement in both lungs on chest CT, clinical remission of respiratory symptoms, and two throat-swab samples negative for viral RNA obtained at least 24 h apart. In total, 99 patients (61 pneumonia and 38 severe pneumonia) with key information in their medical records were included in this study. Demographic, clinical, laboratory, and treatment data were extracted from electronic medical records. Risk factors that affect disease severity and length of hospital stay were investigated with appropriate statistical methods using R (V3.6.1) or GraphPad Prism (V8.2.1).

To explore the risk factors associated with disease severity, univariate and multivariate logistic regression models were used. In univariate analysis, hypertension, lymphopenia, elevated neutrophil count, lactate dehydrogenase (LDH), C-reactive protein (CRP), and symptoms such as dyspnea, fatigue, and anorexia/lethargy were all associated with severe cases (Fig. 1a; *p* < 0.05). In the multivariable logistic regression model, we included 90 patients (56 pneumonia and 34 severe pneumonia) with complete data for those significant variables from univariate analysis. We identified hypertension (Odds ratio: 3.59; *p* = 0.048), increased CRP (Odds ratio: 1.38; *p* = 0.01) and lymphocyte count (Odds ratio: 0.10; *p* = 0.004) as independent predictors of severe pneumonia (Fig. 1b).

**Fig. 1:**
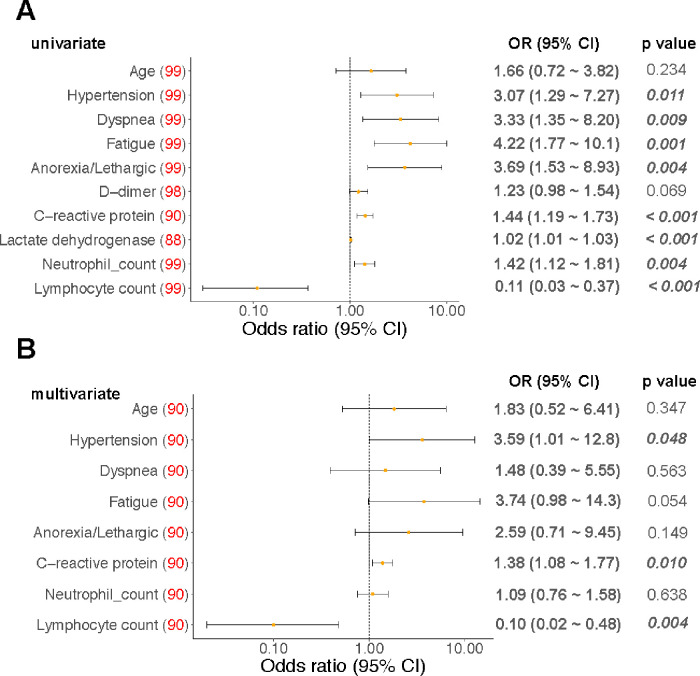
Risk factors associated with disease severity in COVID-19 patients. Univariate (**a**) and multivariate (**b**) logistic regression analysis in COVID-19 patients. OR (odds ratio), 95% CI (confidence interval) and p values are shown. Numbers in red are the number of patients considered for each variable.

In this cohort, all the patients were discharged. As expected, COVID-19 patients with pneumonia were discharged sooner than those with severe cases (Fig. 2a, *p* = 0.016). The median length of hospital stay for pneumonia patients was 22 days, ranging from 9 to 46; while in severe pneumonia patients, it was 25, ranging from 14 to 44. To investigate whether there were any demographic, laboratory and treatments associated with length of hospital stay, log-rank test or Spearman's Rank-Order correlation was used as appropriate (summarised in Fig. 2b). We found length of hospital stay in COVID-19 patients with lymphopenia was prolonged (Fig. 2c, *p* = 0.027). In addition, glucocorticoids use in COVID-19 patients caused prolonged length of hospital stay (Fig. 2d, *p* = 0.002).

**Fig. 2:**
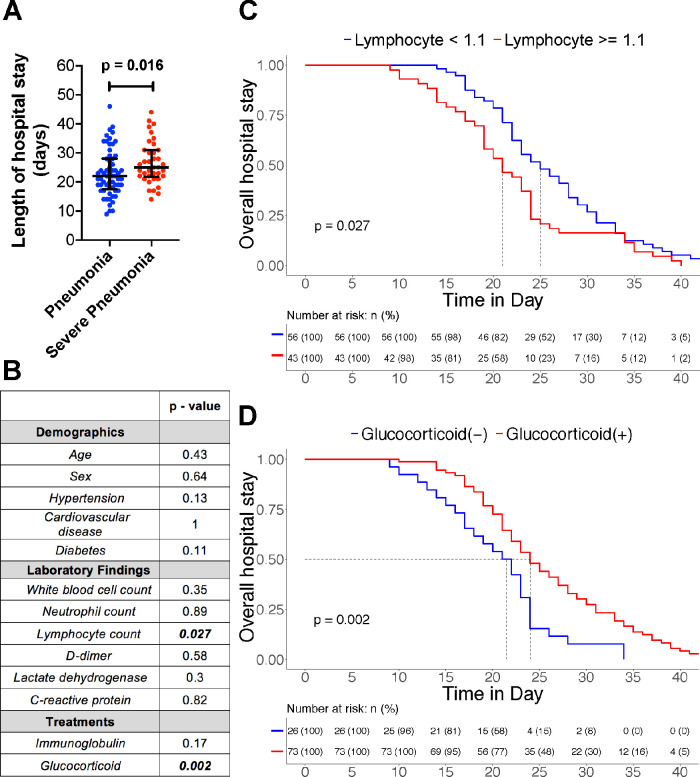
Risk factors associated with length of hospital stay in COVID-19 patients. (**a**) Graph showing length of hospital stay (days) in pneumonia or severe pneumonia patients. Data are median and IQR (interquartile range). *P*-value was calculated by Mann-Whitney *U* test. (**b**) Summary of demographic, laboratory and treatments analysed against length of hospital stay in COVID-19 patients. *P-*values were calculated by log-rank test or Spearman's Rank-Order correlation, as appropriate. (**c**) Kaplan-Meier plot showing the overall hospital stay in COVID-19 patients with normal or low lymphocyte count (< 1.1 × 10^9^/L). Numbers below are n (%). *P*-value was calculated by log-rank test. (**d**) Kaplan-Meier plot showing the overall hospital stay in COVID-19 patients treated with or without glucocorticoids. Numbers below are n (%). *P*-value was calculated by log-rank test.

Cytopathic effects and inflammatory response induced by virus as well as viral evasion of host immune responses are thought to play critical roles in disease severity.^2^^,^^3^ Consistent with this, we identified increased CRP and lymphopenia as independent risk factors for disease severity, while lymphopenia is also a risk factor for prolonged hospital stay. As a result, we recommend surveillance of CRP and lymphocyte counts in the early screening of critical illness in COVID-19 patients.

It has been confirmed that SARS-CoV2 utilizes angiotensin-converting enzyme 2 (ACE2) as receptor for viral cell entry. Given that ACE2 levels are increased in hypertensive patients treated with ACE inhibitors (ACEIs) and angiotensin II type-I receptor blockers (ARBs),^4^^,^^5^ Fang and colleagues proposed the hypothesis that ACE2-stimulating drugs could potentially increase the risk of developing severe and fatal COVID-19.^6^ In this study, we found hypertension is a risk factor for severe cases, independent of age and other variables. We managed to trace 34 hypertensive patients in this cohort. We found 1 out of 14 COVID-19 patients with pneumonia and 4 out of 20 COVID-19 patients with severe pneumonia are on ACEIs/ARBs. The impact of ACEIs/ARBs on disease severity is inconclusive due to small number, but it does suggest a larger cohort study is demanded.

The use of corticosteroids in COVID-19 patients is controversial.^7^, ^8^, ^9^ The Chinese Thoracic Society has developed an expert consensus statement on the use of corticosteroids in COVID-19 patients,^10^ in which low-to-moderate dose of corticosteroids in short courses for critically ill COVID-19 patients is recommended. However, there are potential risks associated with corticosteroids, such as secondary infections and prolonged virus shedding.^8^ In our study, the log-rank test suggested glucocorticoids use led to a prolonged length of hospital stay in COVID-19 patients, which discourages its use.

There are several limitations in this study. First, not all laboratory examinations were done in all patients due to the retrospective nature of this study. In addition, interpretation of our findings might be limited by the sample size. Despite these limitations, with appropriate statistical tools we are able to identify several risk factors to predict disease severity and length of hospital stay in COVID-19 patients.

Taking together, we report hypertension, increased CRP and lymphopenia as independent risk factors for disease severity. COVID-19 patients with lymphopenia have longer length of hospital stay. In addition, our data do not support corticosteroid treatment for COVID-19 patients.

## Declaration Competing of Interest

None.
